# Notes and News

**Published:** 1893-11-18

**Authors:** 


### Notes and News.

Collected and Communicated.

The late Sir Andrew Clark, Bart., was an honour to the medical profession whilst he lived, and the profession received a public recognition in his person at the funeral service in Westminster Abbey on Saturday which was unexampled. The Royal College of Physicians was the first to promote a memorial to the Dean of Westminster, but that memorial was signed by the leaders of both political parties in the House of Commons, *i.e.*, by Mr. Gladstone and Mr. A. J. Balfour, by the Speaker of the House, and by eminent men representing many professions and classes. There have been many public funerals in Westminster Abbey, but the ceremony on the 11th inst. was unique in the circumstance that every person present, with one or two conspicuous exceptions, was evidently there from his personal regard and affection for the dead. Sir Andrew Clark suffered in his lifetime because it was his wont to appear in public in support of causes outside the medical profession which he believed to be good and noble. This service in Westminster Abbey proves to demonstration that Sir Andrew was right in the course he thus took, and we venture to hope the fact will be recognised and acted upon in future by the leaders of the medical profession.

We think it was fitting that Sir Andrew Clark should be buried, not in Westminster Abbey, but in the country. Under the spreading branches of a magnificent cedar tree—the largest, we believe, in the country—Sir Andrew Clark was laid to rest in the picturesque churchyard of St. Mary's, Essendon, Herts. About a hundred colleagues and friends went down to Essendon in order to be present at the funeral, and all were struck by the beauty of the surrounding scenery, and by the magnificent sunset which lit up the whole scene and made all realise the charm of the undulating woodland scenery with its autumn tints. Those present were deeply touched, but it was matter for regret that Sir Andrew Clark's favourite hymn, instead of being sung to its own familiar tune, was set to one so inappropriate as to jar on the feelings of all present. The presence of such a company was a tribute to the affection which the deceased inspired amongst his contemporaries, and all who had the privilege of knowing him intimately. Not the least touching evidence of the affectionate regard in which Sir Andrew Clark was held is afforded by the circumstance that the wreaths which remained on the coffin as it was lowered into the grave were one from the Princess Louise, a second bearing these words, "Hawarden flowers from Mr. and Mrs. Gladstone," and a third sent by the servants, who will sadly miss so good and kind a master.

It is not pleasant hearing for the dwellers in the West-end that small-pox has declared itself there. Kensington is, so far, the most affected district, and a sufficient number of cases have been removed from there to the hospitals to regard the disease as epidemic. In spite of the opening of the new hospital at Tooting, the fever patients are still inadequately provided for. Whilst this is the case there is little likelihood of stamping out scarlet fever from the metropolis. In Shoreditch, a much affected district, the guardians do not approve of the present method of selection of such as have prior claim to admission into the hospitals. The selection rests with the Medical Officer of Health, whereas the guardians hold that patients should be admitted in direct ratio to their poverty.

Further efforts to release Dr. Cornelius Herz from custody were made by Sir George Lewis last week at Bow Street. It is felt by his medical attendants that a decision as to his culpability or innocence would tend towards his long-delayed recovery. Since January last Dr. Herz has been too ill to leave Bournemouth, and previous efforts and those recently made by Sir George Lewis have failed to effect any arrangement whereby his trial could take place either at Bournemouth or elsewhere, in his absence. Sir John Bridge, to whom the appeal at Bow Street was made, did not seem to think the English and French medical evidence sufficiently co-incident to warrant him in accepting the opinion that Dr. Herz was wholly unfit to travel. Under the circumstances Dr. Herz will have to decide between the fears of his doctors and his own anxiety to receive a verdict.

Whilst the case of Dr. Herz is one of great interest in England it is one of far-reaching importance on the other side of the water. He was the cause of a very stormy scene at the Academy of Medicine in Paris. Professor Brouardel came prepared to read his report on the last consultation to the meeting. This the Academy altogether refused to hear. They assumed the attitude that professional secrecy should not in any case be violated, and that as a professional body they had only to regard individuals as private patients, and in no wise allow their conduct to be affected by political issues. This attitude Dr. Brouardel himself advocates in one of his own works, and this may have induced him to withdraw his report and thus prevent a more regrettable termination to the scene.

